# Combined Micellar Liquid Chromatography Technique and QSARs Modeling in Predicting the Blood–Brain Barrier Permeation of Heterocyclic Drug-like Compounds

**DOI:** 10.3390/ijms232415887

**Published:** 2022-12-14

**Authors:** Małgorzata Janicka, Anna Śliwińska, Małgorzata Sztanke, Krzysztof Sztanke

**Affiliations:** 1 Department of Physical Chemistry, Faculty of Chemistry, Institute of Chemical Science, Maria Curie-Skłodowska University, 20-031 Lublin, Poland; 2 Doctoral School of Quantitative and Natural Sciences, Maria Curie-Skłodowska University, 20-038 Lublin, Poland; 3 Department of Medical Chemistry, Medical University of Lublin, 4A Chodźki Street, 20-093 Lublin, Poland; 4 Laboratory of Bioorganic Compounds Synthesis and Analysis, Medical University of Lublin, 4A Chodźki Street, 20-093 Lublin, Poland

**Keywords:** micellar chromatography, QSARs, log *BB*, lipophilicity, heterocyclic drug-like compounds

## Abstract

The quantitative structure–activity relationship (QSAR) methodology was used to predict the blood–brain permeability (log *BB*) for 65 synthetic heterocyclic compounds tested as promising drug candidates. The compounds were characterized by different descriptors: lipophilicity, parachor, polarizability, molecular weight, number of hydrogen bond acceptors, number of rotatable bonds, and polar surface area. Lipophilic properties of the compounds were evaluated experimentally by micellar liquid chromatography (MLC). In the experiments, sodium dodecyl sulfate (SDS) as the effluent component and the ODS-2 column were used. Using multiple linear regression and leave-one-out cross-validation, we derived the statistically significant and highly predictive quantitative structure–activity relationship models. Thus, this study provides valuable information on the expected properties of the substances that can be used as a support tool in the design of new therapeutic agents.

## 1. Introduction

The development of new drugs with desired properties is a tedious, laborious, time-consuming, and expensive process. Quantitative structure–activity relationship (QSAR) methods should be useful tools on this complicated path. The approach is based on the assumption that biological (pharmacokinetic) properties of structurally similar compounds can be quantitatively described by mathematical models. In addition, these models should predict with good probability the activity of structural analogs not yet synthesized. Although the establishment of QSAR models involves a number of steps and conditions, such as use of reliable and accurate input data, selection of relevant descriptors, and use of appropriate software and validation of the suggested model, the advantages of the QSAR methodology are not in doubt [1,2,3,4,5,6,7,8]. First of all, it reduces overhead costs, decreases the time of obtaining positive results, reduces animal testing, and respects the principles of Green Chemistry [5].

A crucial factor for a drug candidate is transport through the blood–brain barrier (BBB). Satisfactory transport through the BBB is an essential prerequisite for a potential drug to affect the central nervous system. However, to avoid side effects, the agents that act peripherally should not cross the BBB. In both cases, the permeability of the BBB must be known and should be evaluated at the earliest possible stage of testing [3,4,5,9,10,11,12].

The uptake of a compound into the brain is a complex process [13,14,15,16]. However, it is known that moderately lipophilic drugs can cross the BBB by passive diffusion [17,18,19,20,21]. A measure of the ability of a compound to penetrate the BBB is defined as the log *BB*, which is the ratio of the concentration of the drug molecules in the brain to the concentration in the blood at equilibrium [3]. The traditional method of measurement, requiring animal or human testing, is time-consuming, expensive, and complicated. For this reason, it is unacceptable in screening, and the indirect methods for log *BB* prediction are highly desired. QSAR methods combining in vitro and in silico techniques have become very attractive alternatives to in vivo testing [9]. Generally, QSAR models are functions of a molecule’s structure, electronic properties, and lipophilicity. More or less sophisticated models using new descriptors selection have been proposed [22,23,24,25,26,27,28,29,30,31,32]. As polarity descriptors are used polar surface area (*PSA*) or topological polar surface area (*TPSA)* [33,34], the numbers of hydrogen bond donors (*HBD)* and hydrogen bond acceptors (*HBA)* [4,35,36]. Molecular size is characterized by molar weight, polarizability parameter, parachor, refractivity, molar volume, etc. [7,10]. Lipophilicity is usually evaluated using the log *P*_o/w_ parameter describing solute partitioning between water and *n*-octanol.

For many years, various liquid chromatography techniques have been known and accepted to predict the lipophilic properties of organic substances, especially bioactive ones, as an alternative for the shake-flask technique. The theoretical bases are Collander-type equations [37], confirming good relationships between log *P*_o/w_ (the logarithm of partition coefficient in the *n*-octanol/water system) and chromatographic parameters. In particular, the log *k*_w_ parameter corresponding to the retention of the solute in water as the mobile phase is considered as the lipophilicity descriptor [37,38,39,40,41,42]. 

Chromatographic techniques assessing the lipophilic properties of substances contribute to the development of QRAR (quantitative retention–property relationship) and/or QSAR (quantitative structure–activity relationship) models for predicting the penetration of substances through the BBB. There are different reversed-phase liquid chromatography techniques, both planar and column [37,38,39,40,41,42,43,44,45], used in this type of research. Chromatography with stationary phases that imitate biological partitioning systems, such as an artificial membrane, phases with immobilized lipids, albumin, cholesterol, ceramides, or liposomes, allow not only the prediction of the lipophilic properties [37,38,39,40,41,42,43,44,45] but also the behaviors of solutes in real biological systems (bounding to serum albumin, skin permeation, blood–brain barrier permeability, intestinal absorption, concentration of unbound form in blood, and others) [46,47,48,49,50,51].

Similar possibilities are offered by micellar liquid chromatography (MLC) using surfactants as components of the mobile phase. MLC is using a surfactant solution above the critical micellization concentration (*cmc*). Under these conditions, the micelles form the so-called micellar pseudophase in the bulk phase. The surrounding bulk water or aqueous–organic mixture contains surfactant monomers in a concentration approximately equal to the *cmc*. Moreover, surfactant monomers modify the surface phase as a result of the hydrophobic interactions between the tail of the surfactant and the alkyl chain grafted to the carrier surface of the stationary phase. Molecular interactions present in this system, i.e., solute association with the polar head of the surfactant, solute penetration into the micelle core, and solute interactions with adsorbed surfactant and alkyl chains, affect retention by three different equilibria, which are (1) the solute distribution between the micelle (micellar pseudophase) and the bulk phase, (2) the solute partition between the stationary phase modified by the surfactant and the bulk phase, and (3) the direct transfer of solute molecules between the surfactant-modified surface and the micelle [52,53,54,55,56].

The effect of the concentration of the surfactant in the effluent on the retention of the solute can be described by Foley’s equation [57], where the following relationship exists between the retention parameter, *k*, and the concentration of the surfactant in the effluent:

(1) $$\frac{1}{k}=\frac{1}{k_{m}}+\frac{K_{AM}}{k_{m}}\left[M\right]$$

where [*M*] is the total concentration of surfactant in the mobile phase minus the critical micellization concentration, *cmc*, *K*_AM_ is the constant that describes solute–micelle binding, and *k*_m_ is the solute retention parameter at zero micellar concentration, i.e., at surfactant monomer concentration equal to *cmc*. The *K*_AM_ and *k*_m_ parameters can be evaluated from the slope and intercept of experimental 1/*k* vs. [*M*] relationships. Equation (1) describes a linear dependence with decreasing retention as the micelle concentration increases. This equation is valid for aqueous solutions of surfactant or mobile phases with the same concentrations of the organic modifier. The micellar retention parameter, log *k*_m_, is considered analogous to the log *k*_w_ value evaluated in RPLC. Thus, this parameter is considered as a lipophilicity descriptor, and Equation (1) is a simple way to achieve the indirect determination of the lipophilic properties of compounds. It is postulated that retention in micellar chromatography depends on the hydrophobic (lipophilic), electronic, and steric features of the compounds in a similar way as many pharmacokinetic phenomena. Additional similarity is indicated by the fact that the phospholipids, cholesterol, fatty acids, and triglycerides that are present in the extracellular and intracellular fluids also form micelles with proteins.

All the compounds (**1**–**65**) [58,59,60,61,62,63,64,65,66,67,68,69,70,71,72,73,74] investigated in present studies have been designed and synthesized in our laboratory. Their molecular structures—confirmed by spectroscopic methods in previous studies [58,59,60,61,62,63,64,66,67,68,69,70,71]—are presented in Table 1. The purity and homogeneity of all the compound samples (**1**–**65**) have been proven [75]. These heterocyclic molecules may be of pharmaceutical importance due to their promising anticancer [58,59,60,61,62,63,64,65,66,67,68,70], analgesic [58,59,69,71], antiviral, and antihemolytic [65] activities as well as their medical applicability [61,62,63,67,68,69,73,74], high purity, and thermal stability [72]. Compounds belonging to the particular structurally related classes, e.g., I (**1**–**6**), III (**12**–**17**), IV (**18**–**28**), V (**34**, **37**–**40**), and VII (**61**–**65**), have been recognized as anticancer agent candidates, especially in the treatment of human cancers of the cervix, breast (**1**–**6**, **12**–**17**, **18**–**28,** and **61**–**65**) [60,61,62,63,64], lung, ovary (**1**–**6** and **61**–**65**) [60,61,62], pharynx, tongue (**1**–**6**) [65], and in the therapy of human multiple myeloma (**12**–**17**, **34**, **37**–**40**) [63,64,70]. Simultaneously, most of these molecules — as promising drug candidates — proved to be low in toxicity to normal cells, including erythrocytes [60,61,62,63,64,65,66,67,68,70]. Compounds **1**–**3** were found to be safer for the early life stages of zebrafish than an antiviral agent acyclovir, while molecules **3**–**6** were equally safe as this pharmaceutic [65]. Additionally, all these heterocycles (**1**–**6**) showed a protective effect on oxidatively stressed red blood cells, which was stronger or comparable to that of ascorbic acid. It has been shown that molecule **6** is antivirally active, too [65]. Furthermore, many compounds with low in vivo toxicity (**8**–**11**, **32**, **34**, **39**, **42**, **48**, **51**, and **53**) were found to be analgesic active, with three structures (**8**, **42**, and **51**) showing the strongest antinociceptive activity in the acetic acid-induced writhing test in mice [58,59,69,71]. The first two analytical procedures for quantifying the pharmacologically relevant molecules (**15** and **39**) in solution and in serum samples have recently been developed and disclosed [73,74].

In our investigation, for the first time, the lipophilic properties of all the compounds (**1**–**65**) were experimentally determined by micellar liquid chromatography (MLC). The micellar lipophilicity parameters from MLC (as an experimental in vitro technique) and in silico data were combined with the aim of building the most satisfactory models for the prediction of the BBB permeation of 65 heterocyclic drug-like compounds. The linear quantitative structure–activity relationship models were produced using multiple linear regression on a database that consisted of different lipophilicity, polarity, electronic, and molecular size descriptors. The predictive ability of the developed models was validated by leave-one-out cross-validation (LOOcv).

## 2. Results

Chromatographic retention parameters (*k*) were calculated according to the following relationship:

(2) $$k=\frac{t_{R}-t_{M}}{t_{M}}$$

where *t*_R_ and *t*_M_ are retention times for a given solute and an unretained compound, respectively.

**Figure 1:**
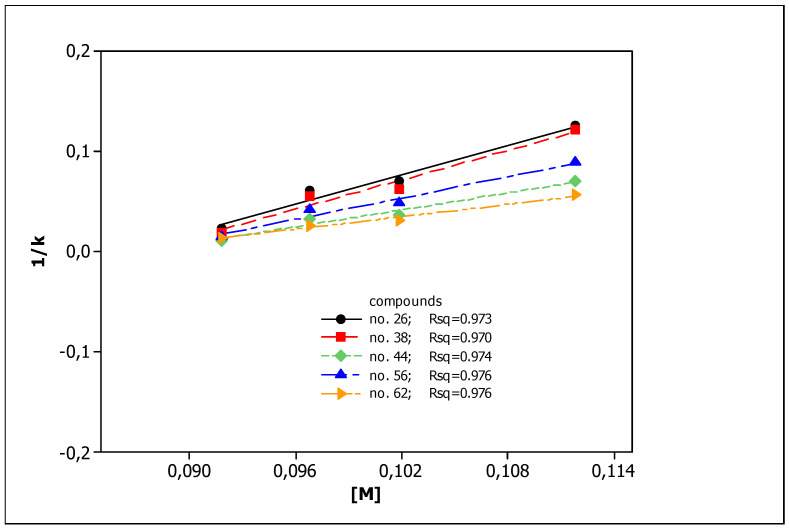
The 1/*k* vs. [*M*] relationships obtained for compounds **26**, **38**, **44**, **56**, and **62** from MLC measurements.

To describe the effect of surfactant concentration on solutes retention, we applied the Foley equation and four effluents with different SDS concentrations: 0.1, 0.105, 0.11, and 0.12 mol L^−1^. The obtained results are presented in Figure 1 as the 1/*k* vs. [*M*] relationships (Equation (1)) for five chosen compounds. The parameters of Equation (1) for all compounds tested, together with coefficients of determination *R*^2^, are presented in Table 2.

Physico-chemical parameters characterizing the investigated compounds, i.e., the logarithm of the partition coefficient (log *P*_o/w_) in the *n*-octanol/water system, the numbers of hydrogen bond donors (*HBD*), acceptors (*HBA*), and rotatable bonds (*NRB*), molecular weight (*MW*), topological polar surface area (*TPSA*), polarizability (*α*), and parachor (*Ƥ*), are presented in Table 3. Also included are the log *BB* parameters. All the values were evaluated in silico from molecular structures (ACD Percepta software). The log BB* parameters from Table 3 were calculated in our previous studies [75], using the equation derived for 23 structurally similar compounds with known experimental log *BB* values. The relationship between both, log *BB* and log BB*, parameters is linear and very good (*R*^2^ = 0.9010).

## 3. Discussion

### 3.1. Chromatographic Data

**Figure 2:**
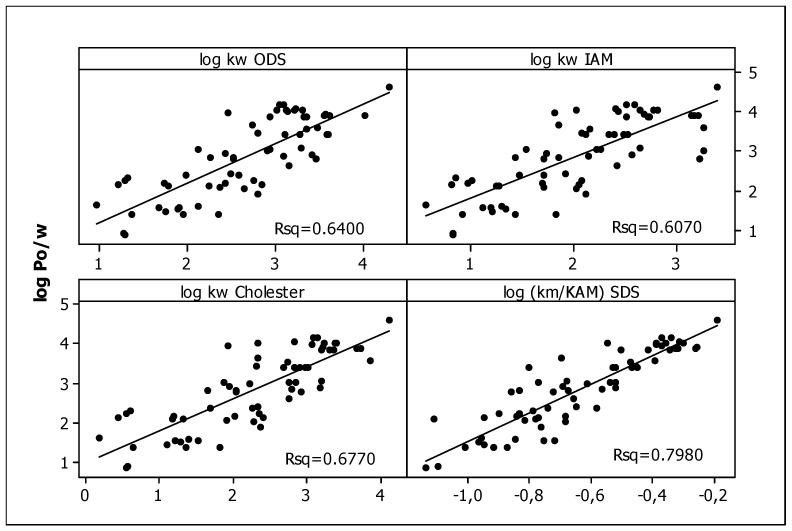
The relationships between log *P*_o/w_ and log *k*_w, ODS_, log *k*_w, IAM_, log *k*_w_, _Cholester_ [75], and log (*k*_m/_K_AM_) values.

In our previous studies [75], all the compounds presented in Table 1 were analyzed using RP HPLC with three different stationary phases imitating biological partitioning: ODS (octadecylsilyl), IAM (artificial immobilized membrane), and Cholester (immobilized cholesterol). As lipophilicity descriptors, log k_w_ parameters were used, describing solute retention in the system with 100% aqueous mobile phase, calculated by linear extrapolation. Presently, to mimic biodistribution, we have used the micellar chromatography technique with SDS as the mobile phase component and the Foley equation to describe solute retention. Very good linear relationships (R^2^ > 0.9) were obtained for all tested compounds (Figure 1, Table 2), confirming that the Foley equation correctly describes the retention of solutes in the tested chromatographic systems. Unfortunately, due to the strong retention of the tested compounds, the intercepts for all equations are negative. This is inconsistent with the physico-chemical interpretation of the regression coefficient of this equation: the intercept is equal to the reciprocal of k_m_, i.e., the retention factor in the system in which the concentration of unbound surfactant ([M]) in the effluent is equal to zero. This value may in no case be less than zero. For this reason, we decided to use log (k_m_/K_AM_) values calculated from the slopes of Equation (1) as micellar lipophilicity descriptors of the compounds. The rationale is that both parameters (k_m_ and K_AM_) characterize lipophilic properties of solutes: their affinity to the stationary phase modified by the surfactant (k_m_) and binding to the micelles (K_AM_). Moreover, in our previous research [76] on the group of pesticides, we compared both micellar parameters (log k_m_ and log K_AM_), obtaining a very good rectilinear relationship with R^2^ = 0.9724. To assess the correctness of our deductive reasoning, we examined the correlation between log (k_m_/K_AM_) values and partition coefficients log P_o/w_ obtained in silico from molecular structures of compounds, commonly accepted as lipophilicity descriptors. They were compared with analogous relationships for other chromatographic lipophilicities (log k_w_) evaluated for ODS, IAM, and Cholester columns. The graphs presented in Figure 2 show the correct (direct proportion) relationships with moderate fit (R^2^ > 0.6) but the best one for the micellar parameter (R^2^ = 0.7980). The correlations between different chromatographic parameters considered as lipophilicity descriptors (log k_w_ and log (k_m_/K_AM_) are also moderate—R^2^ in the range 0.6002–0.6990. The above relationships confirm that micellar parameters can be considered as lipophilicity descriptors.

### 3.2. In Silico Data

The compounds investigated have in silico log *BB* values in the range −0.293–0.712 (Table 3), and they penetrate the blood–brain barrier better (log *BB* > 0) or weaker (log *BB* < 0). Without more in-depth research, it is impossible to decide which may be CNS-active. It is clear that CNS activity requires BBB permeation, but some drugs that are not CNS-active may still pass through the BBB and show no activity because they do not interact with any CNS targets. Similarly, some drugs with an expected peripheral site of action may pass through the BBB, leading to undesirable side effects on the CNS. Molar weights of compounds range from 242.28 to 387.26 g mol^−1^ (Table 3) and meet the rule formulated by Lipiński and coworkers (The Rule of 5, Ro5) [77], i.e., *MW* ≤ 500 g mol^−1^ or one of the “Rules of Thumb” proposed by Clark [78] (*MW* ≤ 450 g mol^−1^) for brain permeation by drugs and clinical candidates. The numbers of hydrogen bond acceptors *HBA* ≤ 10, and the numbers of hydrogen bond donors *HBD* ≤ 5. HBDs also fulfill Ro5. The investigated molecules are bases: *HBD* = 0 for all compounds [75], with *HBA* ranging from five to eight (Table 2). Moreover, the polar surface areas, described by *TPSA* values, are in the range 48.27–57.50 Å^2^, which meets the next principle given by Clark [78]. For good brain permeation, the polar surface area of the compound should be below a certain limit. In the literature on the subject, there are two differing limits: 90 Å^2^ suggested by van de Waterbeemd et al. [36] and a lower limit of 60–70 Å^2^ proposed by Kelder et al. [33]. The test substances (except compounds from groups II and III) satisfy both limits. Table 3 also provides values of parachor (*Ƥ*) ranging from 497.81 to 763.55 m^3^ mol^−1^ and polarizability (*α*) ranging from 27.55 to 41.70 Å^3^.

**Figure 3:**
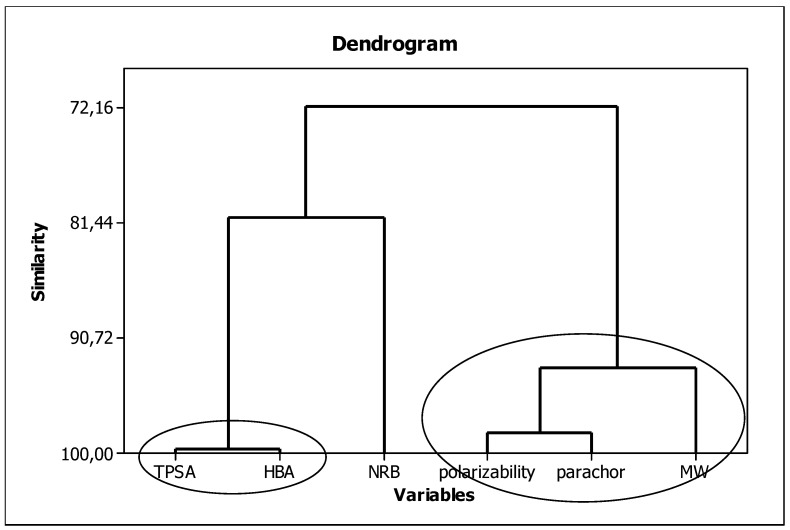
Similarities between the in silico molecular descriptors.

In our procedure, parameters characterizing the lipophilic, structural, and electronic properties of molecules, i.e., micellar parameter (log *k*_m_/*K*_AM_), *MW*, *TPSA*, *HBA*, *α*, and *Ƥ*, will be used as independent variables. To ensure the variables have a minimal impact on each other (to keep the principle of orthogonality), we checked for similarities among them. The results are presented in Figure 3. Here, we can see three groups of strongly correlated descriptors: (I) including *TPSA* and *HBA* (99.62%) characterizing the polar nature of the molecule and its ability to form hydrogen bonds; (II) consisting of polarizability, parachor, and molar weight (93.16%) related to the size of the molecule; and a single-element group (III) containing *NRB* describing molecule flexibility.

### 3.3. Establishment of Quantitative Structure–Activity Relationships

The establishment of QSAR models involves the use of reliable and accurate input data, selection of relevant descriptors, use of appropriate software, and validation of the suggested model [79,80]. Our models have involved descriptors characterizing solutes’ lipophilicity (micellar log (*k*_m_/*K*_AM_) values), polarity (*TPSA*, *HBA*), flexibility (*NRB*), and size (*MW*, *α*, *Ƥ*). We used in silico (*HBA*, *NRB*, *MW*, *TPSA*, *α*, *Ƥ*, log *BB*, and log BB*) and in vitro (log *k*_m_/*K*_AM_) data. The models were produced using the multiple linear regression (MLR) technique on a database that consisted of 65 recently discovered drug-like compounds. The linear quantitative structure–activity relationships (QSARs) were presented for the modeling of log *BB* values. The developed models were validated by leave-one-out cross-validation (LOOcv).

Validation is a necessary step to establish the quality of a QSAR model [81,82,83]. In our investigations, traditional validation metrics were applied: the mean squared error (*MSE*), the coefficient of determination (*R*^2^), the determination coefficient adjusted (*R*^2^_adj_), and the determination coefficient predicted (*R*^2^_pred_). *R*^2^_adj_ is used to compare the goodness-of-fit for regression models that contain differing numbers of independent variables while *R*^2^_pred_ determines how well a regression model makes predictions. These coefficients (*R*^2^, *R*^2^_adj_, *R*^2^_pred_) have values between zero and one, and the closer to one, the more accurate the model. The *MSE* is used to assess the predictive ability and accuracy of the model, and models with small *MSE* values yield more highly reliable predictions. The derived models were compared and assessed by leave-one-out cross-validation (LOO), and the resulting determination coefficient (*Q*^2^LOO) and *PRESS* were calculated (Table 4). *PRESS* is a good estimate of the real prediction error of the model. It assesses a model’s predictive ability and, in general, the smaller the *PRESS* value, the better the model’s predictive ability [84]. The calculated global *PRESS* value must be lower than the sum of the squares of the response values of the total observations (*SS*). This proves that the developed models predict better than chance [83]. A reasonable QSAR model should have *Q*^2^LOO values greater than 0.6 or the ratio of *PRESS*/*SS* smaller than 0.4 [84]. QSAR models are only valid in the domain they were validated [85] so the determination of applicability domain (AD) is of great importance [86]. AD is a space of (physico-chemical) information, on which the model has been developed and for which it is applicable to make predictions for new compounds. In the present work, we used the leverage approach (Williams plot) where the warning leverage, h*, was calculated according to:

(3) $$h*=\frac{3(p+1)}{n}$$

where *n* is the total number of samples in, and *p* is the number of descriptors involved in the correlation [87].

We searched for relationships between solute property (SP), i.e., log *BB*, and its lipophilicity, molar size, and flexibility descriptors, that is:

log SP = a_0_ + a_1_lipophilicity + a_2_d_I_ + a_3_d_II_ + a_4_d_III_ (4)

where a_0_–a_4_ are regression coefficients, and *d*_I_, *d*_II_, and *d*_III_ are molecular descriptors from group I, II, and III, respectively.

Accounting for possible combinations of independent variables (descriptors from groups I, II, and III) for both parameters (log *BB* and log BB*), twelve models denoted as M1-M6 (for log *BB*) and M7-M12 (for log BB*) have been obtained. Table 4 contains the *R*^2^_,_ *R*^2^_adj_, *R*^2^_pred_, *PRESS*, and *VIF* (variance inflation factors) values calculated for these models, which became the basis for their preliminary evaluation leading to the selection of the most promising ones. Very high *R*^2^ values (>>0.8) indicate that all M1-M12 equations are very good for modeling the data included (good feet of dataset). All *R*^2^_pred_ values are >>0.6, indicating high predictive ability of the models. The decrease in the value of *R*^2^_adj_ compared to the values of *R*^2^ ranges from 0.0053 to 0.0099 units. High predictive abilities of the models are also confirmed by small *PRESS* values, which are in the range 0.2904−0.5268. Moreover, the ratios of *PRESS/SS* are smaller than 0.4. Variance inflation factors *VIF* should not exceed five to ensure that descriptors are moderately correlated. Some *VIF* values presented in Table 4 are equal to five or are slightly higher. For this reason, the M1, M3, M4, M7, M9, and M10 models were excluded from further analysis. Of the remaining six models, the most favorable were those with the highest *R*^2^ and *R*^2^_pred_ values and the lowest *PRESS*. The analysis of the calculated statistical parameters leads to the M5, M11, and M12 models as the most satisfactory. The models selected are as follows:

M5: log *BB* = 0.253(0.232) + 0.198(0.091) log (*k*_m_/*K*_AM_) − 0.160(0.023) *HBA* − 0.019(0.016) *NRB* + 0.002(0.000) *Ƥ*; (5)

M11: log BB^*^ = 0.842(0.191) + 0.106(0.075) log (*k*_m_/*K*_AM_) − 0.148(0.019) *HBA* − 0.004(0.012) *NRB* + 0.0004(0.000) *Ƥ*; (6)

M12: log BB^*^ = 0.911(0.149) + 0.103(0.075) log (*k*_m_/*K*_AM_) − 0.158(0.020) *HBA* + 0.002(0.011) *NRB* + 0.001(0.000) *MW* (7)

**Figure 4:**
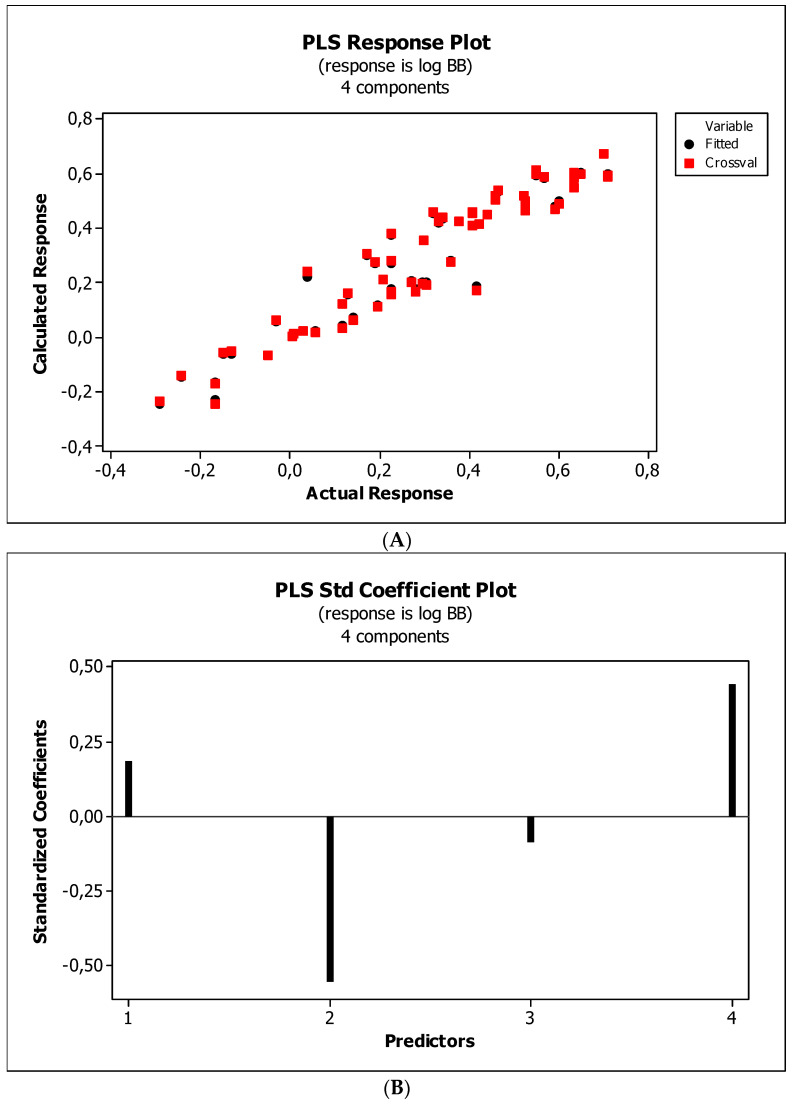

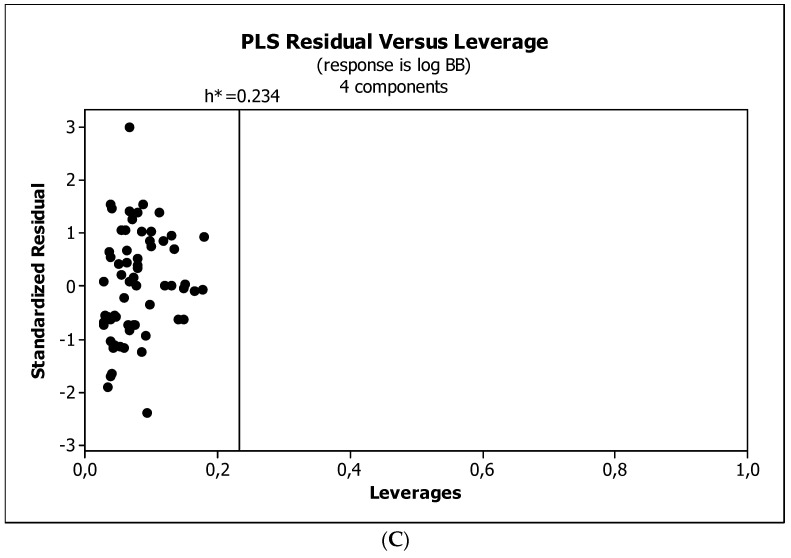
(**A**) Model M5: the response plot. (**B**) Model M5: the standardized coefficients plot. (**C**) Model M5: Williams plot.

**Figure 5:**
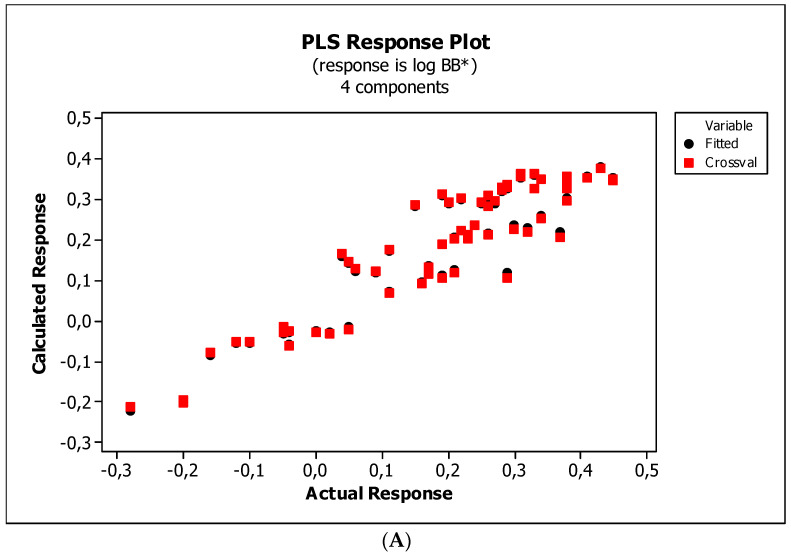

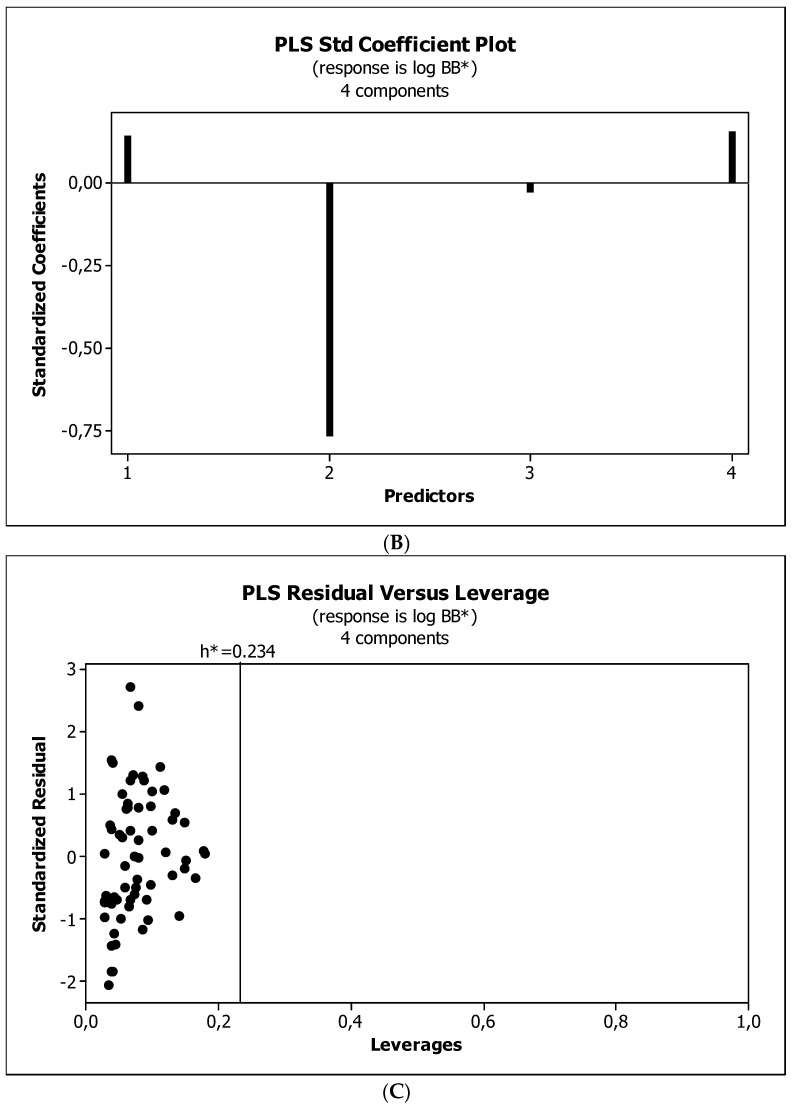
(**A**) Model M11: the response plot. (**B**) Model M11: the standardized coefficients plot. (**C**) Model M11: Williams plot.

**Figure 6:**
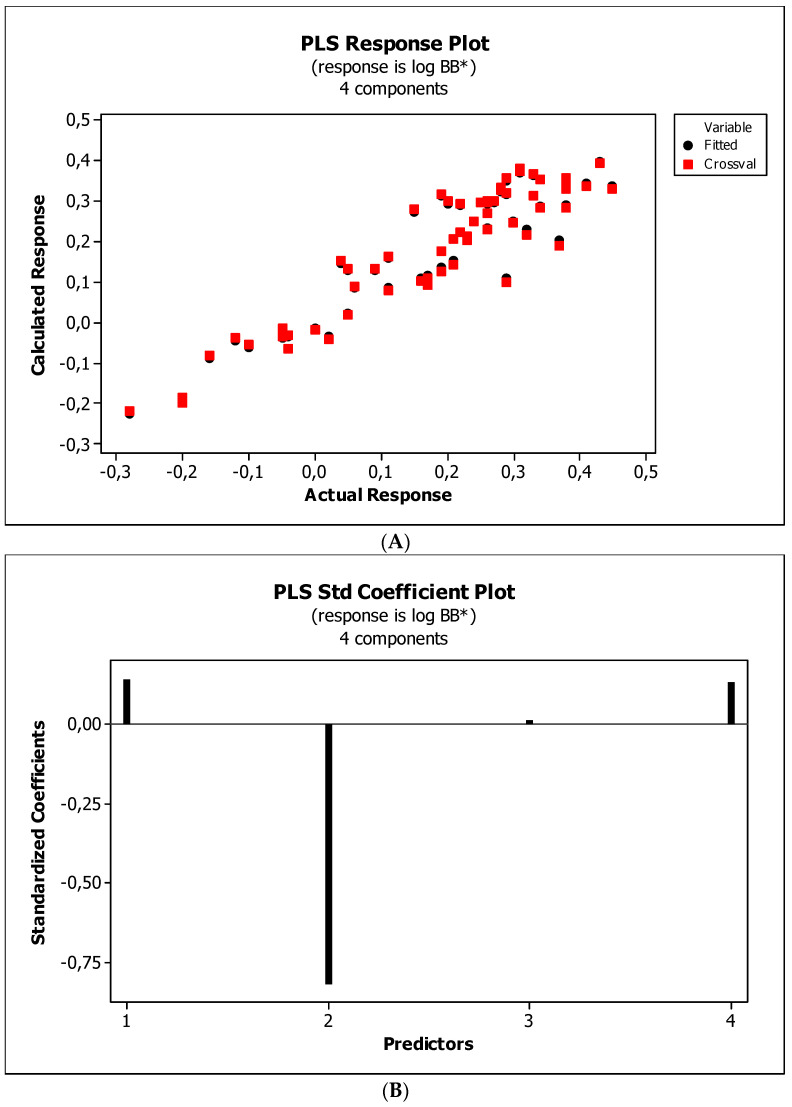

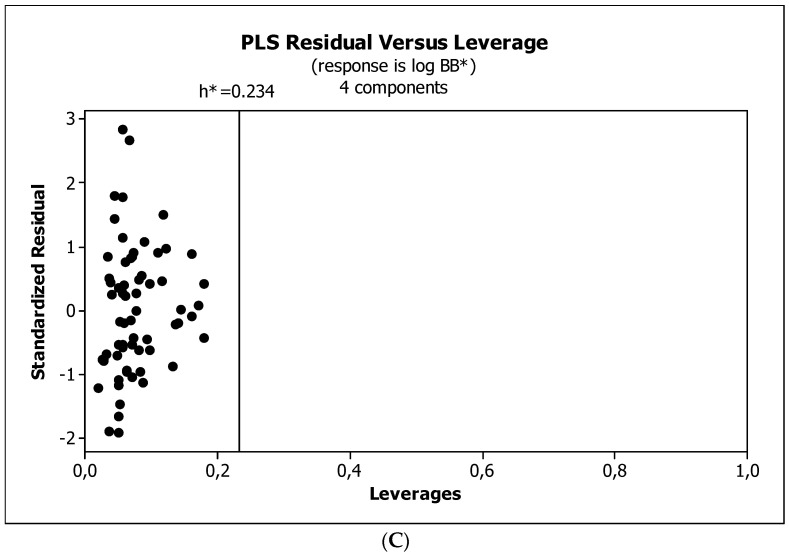
(**A**) Model M12: the response plot. (**B**) Model M12: the standardized coefficients plot. (**C**) Model M12: Williams plot.

Figure 4, Figure 5 and Figure 6 present graphical results of LOO cross-validation of models M5, M11, and M12. The plots present in segments B illustrate the standard coefficients of the equations of selected models (Equations (5)–(7)). They explain both the direction and strength of the impact of a given descriptor on the calculated biological parameter. The correlations shown in segments A illustrate the relationships between the actual and predicted response, i.e., between log *BB* or log BB^*^ values from Table 3, and these predicted by the QSAR models were developed (Equations (5)–(7)). The applicability domain (AD) was also evaluated and visualized as the Williams plots (segments C). The results proved that the obtained models are valid within the domain for which they were developed.

The results (Figure 4B, Figure 5B and Figure 6B) indicate that the *NRB* parameter seems to be of negligible importance in the case of BBB permeation. Therefore, it was checked whether the omission of this descriptor would affect the evaluation of the derived models. The rationale is to capture the most important properties of compounds and build them into the QSAR model without overfitting the data. The results are presented as the following QSAR equations:

M5*: log *BB* = 0.406(0.196) + 0.181(0.091) log (*k*_m_/*K*_AM_) − 0.180(0.016) *HBA* + 0.002(0.000) *Ƥ*; (8)

M11*: log BB* = 0.874(0.159) + 0.102(0.074) log (*k*_m_/*K*_AM_) − 0.152(0.013) *HBA* + 0.0004(0.000) *Ƥ*; (9)

M12*: log BB* = 0.903(0.139) + 0.108(0.069) log (*k*_m_/*K*_AM_) − 0.156(0.013) *HBA* + 0.001(0.000) *MW* (10)

**Figure 7:**
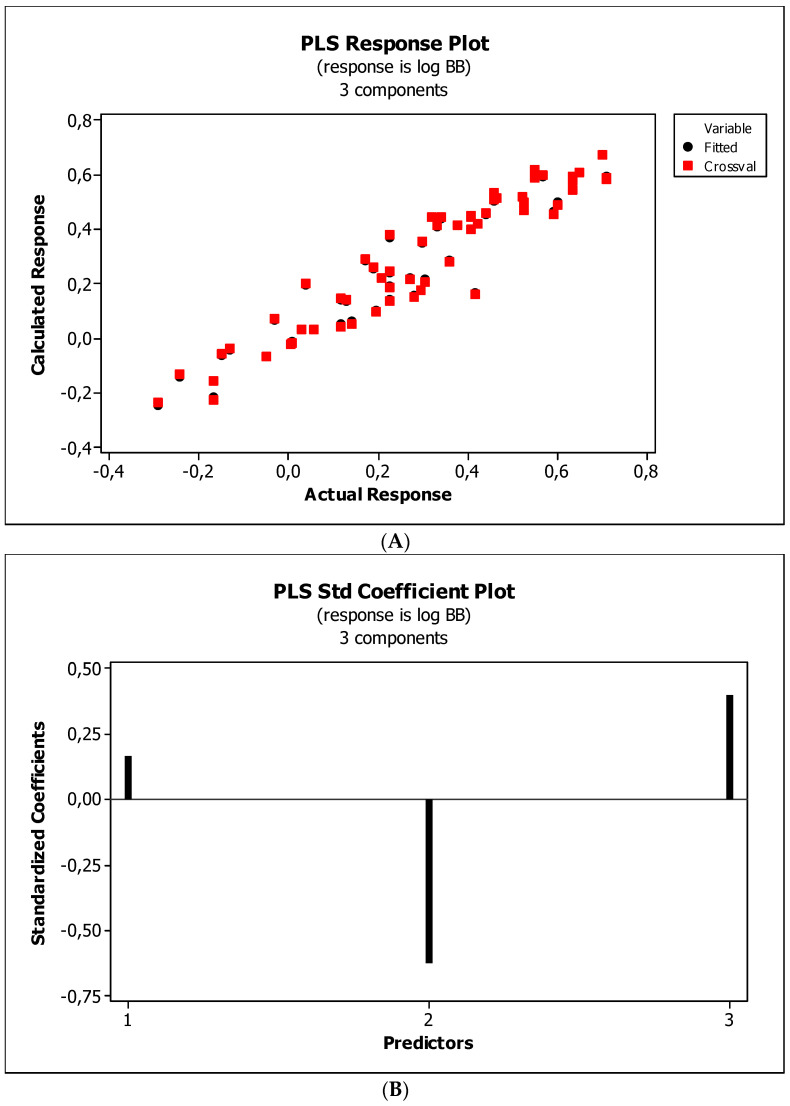

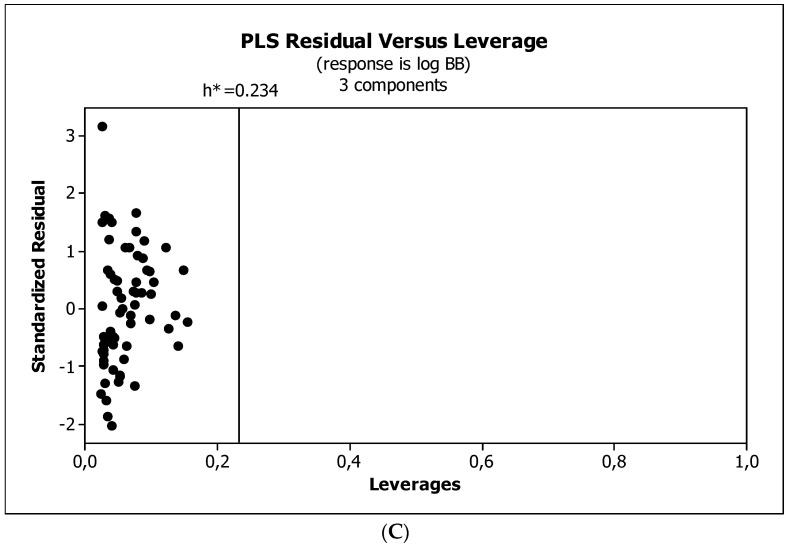
(**A**) Model M5*: the response plot. (**B**) Model M5*: the standardized coefficients plot. (**C**) Model M5*: Williams plot.

**Figure 8:**
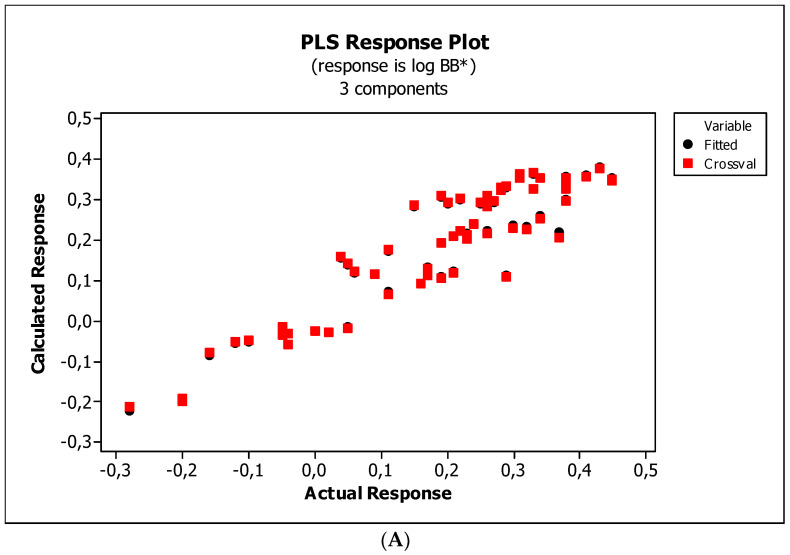

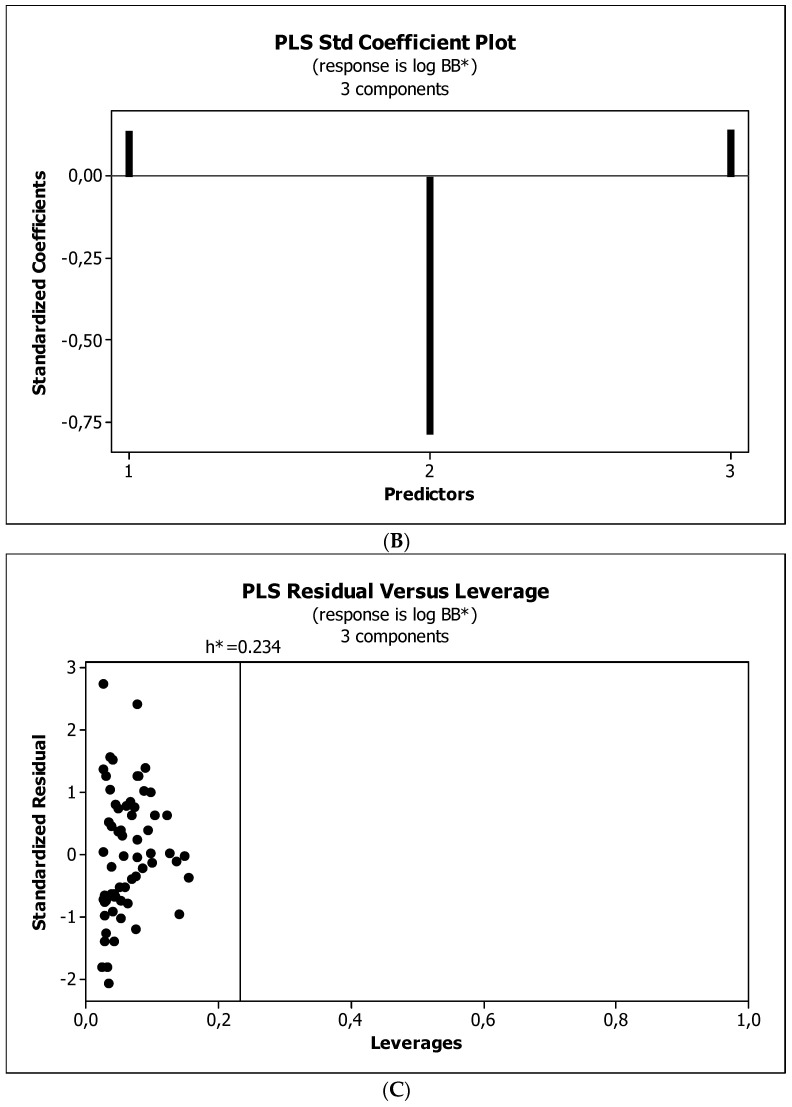
(**A**) Model M11*: the response plot. (**B**) Model M11*: the standardized coefficients plot. (**C**) Model M11*: Williams plot.

**Figure 9:**
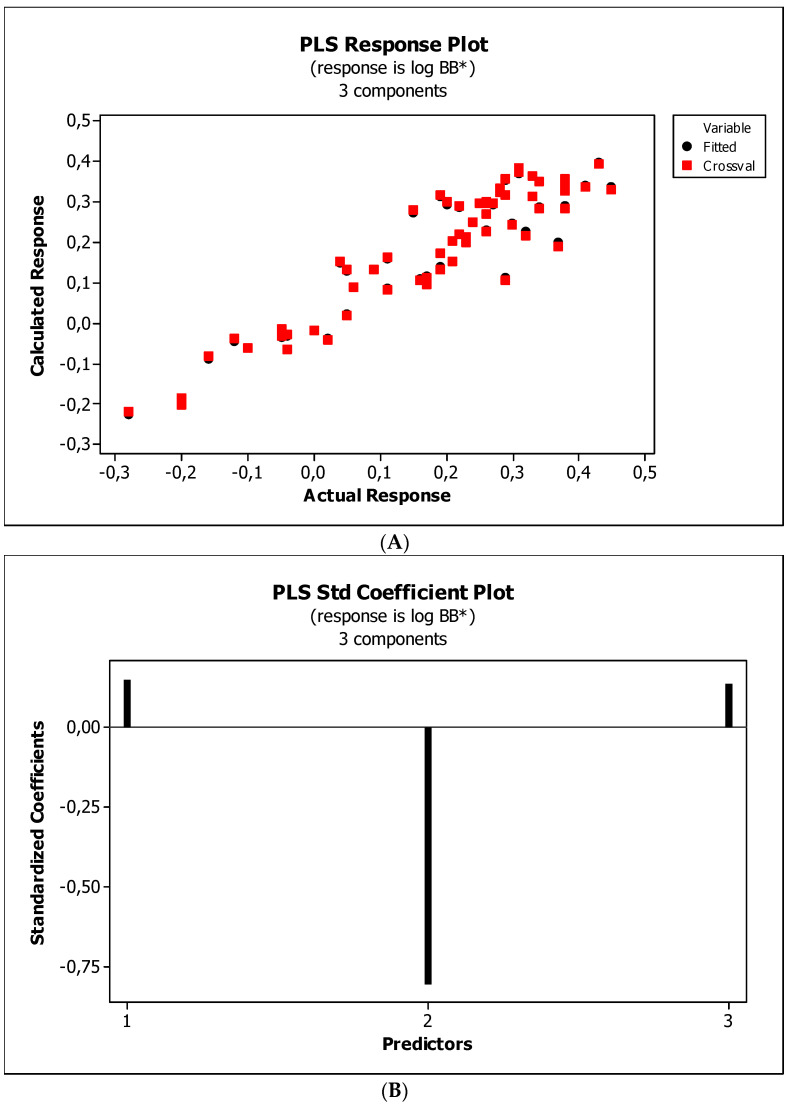

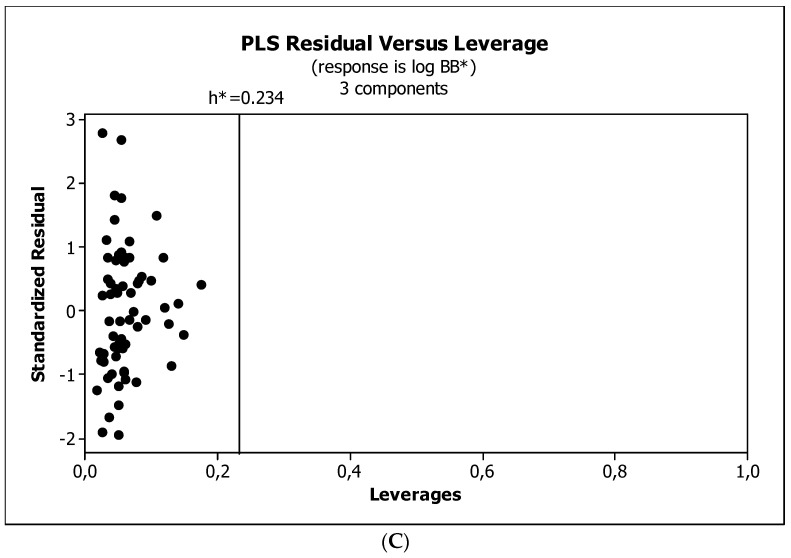
(**A**) Model M12*: the response plot. (**B**) Model M12*: the standardized coefficients plot. (**C**) Model M12*: Williams plot.

The analysis of statistics of the above models, i.e., lower *R*^2^_adj_, *R*^2^_pred_, and *PRESS* values (Table 4), indicates that the new models (omitting the *NRB*) are better for predicting substance permeation through the BBB. The new models were also cross-validated, and the results are presented graphically in Figure 7, Figure 8 and Figure 9.

### 3.4. Interpretation of Descriptors

#### 3.4.1. Lipophilicity

All derived models predict an increase in the BBB permeation with an increase of the substance lipophilicity. Thus, lipophilic compounds have a greater BBB permeability than hydrophilic. However, this influence is not indicated as dominant. Lipophilicity is undoubtedly an important parameter affecting BBB penetration, and it is the base parameter used in different QSARs modeling. Generally, transport of small molecules through membranes occurs via passive diffusion: a molecule dissolves in the phospholipid bilayer, diffuses across it, and then dissolves in the aqueous solution at the other side of the membrane. This process is closely related to the lipophilicity of the molecule: it cannot be too lipophilic, because it will not dissolve in the aqueous environment surrounding the bilayer on both sides; however, it also cannot be too hydrophilic, because it will not penetrate the lipid bilayer. Compounds investigated in this research are moderately lipophilic. Their lipophilicity, as assessed by log *P*_o/w_, ranges from 0.868 to 4.638, for which a positive effect on log *BB* is always expected.

#### 3.4.2. HBA

Our results indicate *HBA* as the dominant factor affecting the log *BB* values. As the number of hydrogen bond acceptors in the molecule increases, its ability to permeate the BBB decreases. Solute polarity and the ability to form hydrogen bonds increase its solubility in the aqueous environment of the membrane, and highly polar molecules do not easily enter the hydrophobic environment of the BBB. In most QSARs models, the dominant descriptor of molecules’ polarity is the polar surface area. Clark [78] and Kelder et al. [33] presented linear regression between log *BB* and *PSA* only, for a group of 45 drugs. In our models, this parameter was also used (M1-M3 and M9), but due to the statistical evaluation the models including *HBA* turned out to be more accurate. However, it should be noted that the *TPSA* values of the test compounds meet the requirements for active substances. The highest *TPSA* value is 83.80 Å^2^, and the number of hydrogen bond acceptors *HBA* does not exceed 10.

#### 3.4.3. Molecular Size

In this study, three descriptors for molecular size were proposed, i.e., molecular weight *MW*, polarizability *α*, and parachor *Ƥ*. In selected models (M5*, M11*, and M12*), *MW* and *α* and *Ƥ* turned out to be the most appropriate. The effect of molecular size on the BBB permeation (log *BB* and log BB* values) is similar to that observed for lipophilicity, i.e., with the increase of *MW* or *Ƥ* values, the permeation of the test substances through the BBB increases. This is in contrast to the results obtained by different researchers who noticed the negative effect of molecule size on compounds’ permeation through biological membranes [88,89,90]. The positive effect of molecular size on the log *BB* (and log BB*) values observed herein could be explained by the partition mechanism of this process. Similarly, Platts et al. [91] obtained the positive effect of molecular size on the permeability through the skin. This relationship is a reflection of the correlation between the size of the molecule and its lipophilicity. It is especially important in the case of low molecular weights (*MW* < 400 mg mol^−1^), such as those in the presented research. Kouskoura et al. [12] noted that moderately increased molecular weight of the compound guarantees that its lipophilicity is sufficient to dissolve in the phospholipid bilayer and enter the BBB via passive diffusion. The same effect was observed in our previous research [92].

#### 3.4.4. Flexibility

Research reveals that blood–brain barrier partitioning is governed not only by solute lipophilicity and polarity but also solute flexibility, solute–membrane flexibility, and solute–membrane binding. In general, ease in traversing the membrane depends on the flexibility of a compound. Limited flexibility can be considered as a merit, and higher flexibility can be proved to be a demerit [93]. The increase in BBB penetration of the solute with increasing solute flexibility was described by Iyer et al. [3]. Veber et al. [94] have found that increasing solute molecular flexibility (measured by the number of rotatable bonds) promotes a decrease in oral bioavailability. It suggests a parabolic relationship between log *BB* and molecular flexibility. That is, some “amount” of flexibility enhances log *BB*, but too much flexibility will diminish log *BB*. Our research (Figure 4B, Figure 5B and Figure 6B) indicates a slight and ambiguous (positive for M12 and negative for M5 and M11 models) influence of the *NRB* value on the penetration of the tested substances through the blood–brain barrier. The numbers of rotatable bonds calculated for tested compounds are in the range from two to six, with average value equal to 3.2. Probably, in the case of the investigated molecules, these values are close to the maximum of the aforementioned parabolic relationship. Ultimately, *NRB* was considered a negligible factor and omitted in subsequent QSAR models.

## 4. Materials and Methods

### 4.1. Reagents and Materials

Isopropanol and acetonitrile (both HPLC grade), as well as sodium dodecyl sulfate SDS (for synthesis), were supplied from Merck (Lublin, Poland). Anhydrous citric acid (C_6_H_8_O_7_) and disodium phosphate (Na_2_HPO_4_)—both pure—were purchased from POCh (Lublin, Poland). Deionized water was produced using the Direct-Q3 UV system (Millipore, Warsaw, Poland).

### 4.2. Instrumental

Shimadzu Vp (Shimadzu, Izabelin, Poland) liquid chromatographic system was used in the measurements. It was equipped with LC 10AT pump, SPD 10A UV–Vis detector, SCL 10A system controller, CTO-10 AS chromatographic oven, and Rheodyne injector valve with a 20 μL loop. A Spherisorb ODS-2 column, 125 × 4 mm i.d., 5 μm (Merck, Lublin, Poland) was applied as the stationary phase.

### 4.3. Chromatographic Conditions

As the mobile phases buffered, SDS mixtures (0.10; 0.105, 0.11, and 0.12 mol L^−1^) with 7% (*v*/*v*) addition of isopropanol were used. The buffer was prepared from 0.01 mol L^−1^ solutions of disodium phosphate and citric acid, and the pH 7.4 value was fixed before mixing with an organic modifier. The flow rate was 1 mL min^−1^. Solutes samples were dissolved in acetonitrile—c.a. 0.005 mg mL^−1^. The compounds were detected under UV light at λ_max_ 254 nm. All measurements were carried out at 25 °C. The dead time values were measured from non-retained compound (e.g., sodium chloride) peaks. All reported *k* values are the average of at least three independent measurements.

### 4.4. In Silico Calculations

Molecular weight (*MW*), topological polar surface area (*TPSA*), polarizability (*α*), parachor (*Ƥ*), the numbers of hydrogen bond donors (*HBD*), acceptors (*HBA*), and rotatable bonds (*NRB*), the partition coefficient in the *n*-octanol/water system (log *P*_o/w_), and the log *BB* values were calculated by ACD/Percepta software, version 1994–2012 (ACD/Labs, Advanced Chemistry Development, Inc., Toronto, ON, Canada).

### 4.5. Statistical Analysis

Linear regression (LR), multiple linear regression (MLR), and leave-one-out cross-validation (LOOcv) were conducted using the statistical software Minitab 16.2.4.0, version 1991–2004 (Minitab Inc., State College, PA, USA).

## 5. Conclusions

The linear quantitative structure–activity relationship models are presented for the modeling and prediction of the BBB permeation of heterocyclic drug-like molecules with promising activity. The models were produced using the multiple linear regression technique on a database that consisted of 65 recently discovered compounds. Among the different lipophilicity, polarity, electronic, and molecular size descriptors that were considered as inputs to the model, four variables were selected, i.e., micellar parameter characterizing the solutes lipophilicity log (*k*_m_/*K*_AM_), the number of hydrogen bond acceptors *HBA* connected with polarity, and parachor or molecular weight (*MW*), describing the molecular size. The rationale was to combine in the model in vitro (micellar lipophilicity parameters log (*k*_m_/*K*_AM_)) and in silico (*α*, *MW*, *Ƥ*) data. The accuracy of the proposed MLR models was illustrated using LOO cross-validation. The predictive ability of the developed models was found to be satisfactory and could be used for designing a similar set of heterocyclic compounds.

Our research confirmed that solute polarity is one of the most important properties affecting the BBB permeation. The increase of *HBA* values decreases the log *BB* (or log BB*) values. In the QSARs models established in our studies, the number of *HBAs* was indicated as the dominant factor. The log *BB* (as well as log BB*) values increase with micellar (chromatographic) log (*k*_m_/*K*_AM_) parameters. This means that more lipophilic drugs have a greater BBB permeability than less lipophilic. This is not the same as their CNS activity, because some compounds that are CNS-inactive may still pass through the BBB and show no activity because they do not interact with any CNS targets. Molecule size descriptors (*MW*, *Ƥ*) also increase the penetration of tested substances through the blood–brain barrier. Generally, polarity, lipophilicity, and molecular size are the most important characteristics of the investigated substances in modeling the permeation of the blood–brain barrier. The results obtained for our heterocyclic compounds indicate that less polar, more lipophilic, and bigger-size molecules partition more readily into the brain.

Our investigation shows the undoubted advantages of micellar chromatography which, combined with computational techniques, enable the prediction of BBB permeation with a high probability. This is an important achievement, especially in screening new potential drugs. It allows the reduction of unethical and expensive animal testing and respects the principles of Green Chemistry.

## Figures and Tables

**Table 1:** Heterocyclic molecules (**1**–**65**) belonging to the particular classes (I–VII).

Class	General Structure	No	R_1_	R_2_
**I**	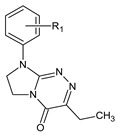	**1**	R_1_ = H	—
		**2**	R_1_ = 4-CH_3_	—
		**3**	R_1_ = 2-Cl	—
		**4**	R_1_ = 3-Cl	—
		**5**	R_1_ = 4-Cl	—
		**6**	R_1_ = 3,4-Cl_2_	—
**II**	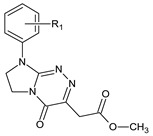	**7**	R_1_ = H	—
		**8**	R_1_ = 4-CH_3_	—
		**9**	R_1_ = 4-OCH_3_	—
		**10**	R_1_ = 4-OC_2_H_5_	—
		**11**	R_1_ = 4-Cl	—
**III**	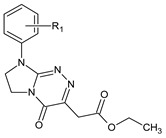	**12**	R_1_ = H	—
		**13**	R_1_ = 4-CH_3_	—
		**14**	R_1_ = 4-OCH_3_	—
		**15**	R_1_ = 3-Cl	—
		**16**	R_1_ = 4-Cl	—
		**17**	R_1_ = 3,4-Cl_2_	—
**IV**	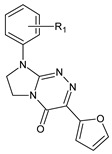	**18**	R_1_ = H	—
		**19**	R_1_ = 2-CH_3_	—
		**20**	R_1_ = 4-CH_3_	—
		**21**	R_1_ = 2,3-(CH_3_)_2_	—
		**22**	R_1_ = 2-OCH_3_	—
		**23**	R_1_ = 4-OCH_3_	—
		**24**	R_1_ = 2-Cl	—
		**25**	R_1_ = 3-Cl	—
		**26**	R_1_ = 4-Cl	—
		**27**	R_1_ = 3,4-Cl_2_	—
		**28**	R_1_ = 2,6-Cl_2_	—
**V**	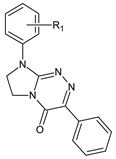	**29**	R_1_ = H	—
		**30**	R_1_ = 2-CH_3_	—
		**31**	R_1_ = 3-CH_3_	—
		**32**	R_1_ = 4-CH_3_	—
		**33**	R_1_ = 2-OCH_3_	—
		**34**	R_1_ = 4-OCH_3_	—
		**35**	R_1_ = 4-OC_2_H_5_	—
		**36**	R_1_ = 2,3-(CH_3_)_2_	—
		**37**	R_1_ = 2-Cl	—
		**38**	R_1_ = 3-Cl	—
		**39**	R_1_ = 4-Cl	—
		**40**	R_1_ = 3,4-Cl_2_	—
**VI**	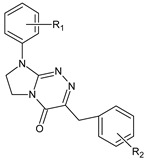	**41**	R_1_ = H	R_2_ = H
		**42**	R_1_ = H	R_2_ = 2-Cl
		**43**	R_1_ = H	R_2_ = 3-Cl
		**44**	R_1_ = H	R_2_ = 4-Cl
		**45**	R_1_ = 4-CH_3_	R_2_ = H
		**46**	R_1_ = 4-CH_3_	R_2_ = 4-CH_3_
		**47**	R_1_ = 4-CH_3_	R_2_ = 3-CH_3_
		**48**	R_1_ = 4-CH_3_	R_2_ = 2-Cl
		**49**	R_1_ = 4-CH_3_	R_2_ = 3-Cl
		**50**	R_1_ = 4-CH_3_	R_2_ = 4-Cl
		**51**	R_1_ = 4-OC_2_H_5_	R_2_ = H
		**52**	R_1_ = 4-OC_2_H_5_	R_2_ = 4-CH_3_
		**53**	R_1_ = 4-OC_2_H_5_	R_2_ = 2-Cl
		**54**	R_1_ = 4-OC_2_H_5_	R_2_ = 3-Cl
		**55**	R_1_ = 4-OC_2_H_5_	R_2_ = 4-Cl
		**56**	R_1_ = 2-CH_3_	R_2_ = 2-Cl
		**57**	R_1_ = 4-Cl	R_2_ = H
		**58**	R_1_ = 4-Cl	R_2_ = 2-Cl
		**59**	R_1_ = 4-Cl	R_2_ = 3-Cl
		**60**	R_1_ = 4-Cl	R_2_ = 4-Cl
**VII**	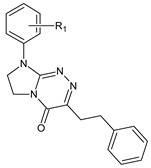	**61**	R_1_ = H	—
		**62**	R_1_ = 4-CH_3_	—
		**63**	R_1_ = 2-Cl	—
		**64**	R_1_ = 4-Cl	—
		**65**	R_1_ = 3,4-Cl_2_	—

**Table 2:** Coefficients of determination and parameters of Equation (1) obtained for the particular compounds.

No	1/*k*_m_	*K*_AM_/*k*_m_	*R* ^2^	No	1/*k*_m_	*K*_AM_/*k*_m_	*R* ^2^
**1**	−0.765	9.066	0.9178	**34**	−0.329	3.829	0.9615
**2**	−0.586	6.826	0.9530	**35**	−0.321	3.685	0.9604
**3**	−0.758	8.907	0.9110	**36**	−0.432	5.006	0.9593
**4**	−0.528	6.157	0.9700	**37**	−0.425	4.933	0.9442
**5**	−0.518	6.028	0.9553	**38**	−0.425	4.870	0.9606
**6**	−0.406	4.722	0.9748	**39**	−0.288	3.326	0.9717
**7**	−1.046	12.573	0.9124	**40**	−0.214	2.470	0.9692
**8**	−0.785	9.294	0.9592	**41**	−0.354	4.119	0.9753
**9**	−1.163	13.801	0.9618	**42**	−0.289	3.322	0.9764
**10**	−0.876	10.274	0.9477	**43**	−0.225	2.944	0.9724
**11**	−0.707	8.316	0.9759	**44**	−0.246	2.822	0.9741
**12**	−0.760	8.902	0.9302	**45**	−0.257	2.976	0.9378
**13**	−0.568	6.593	0.9592	**46**	−0.206	2.361	0.9705
**14**	−0.565	7.520	0.9932	**47**	−0.190	2.198	0.9728
**15**	−0.509	5.898	0.9696	**48**	−0.199	2.272	0.9694
**16**	−0.503	5.825	0.9732	**49**	−0.213	2.448	0.9695
**17**	−0.395	4.569	0.9734	**50**	−0.174	2.001	0.9666
**18**	−0.599	7.079	0.9622	**51**	−0.295	3.399	0.9704
**19**	−0.597	6.995	0.9375	**52**	−0.211	2.441	0.9718
**20**	−0.410	4.836	0.9776	**53**	−0.227	2.606	0.9684
**21**	−0.594	6.814	0.9473	**54**	−0.285	3.202	0.9441
**22**	−0.458	5.274	0.9638	**55**	−0.191	2.203	0.9636
**23**	−0.474	5.694	0.9695	**56**	−0.309	3.549	0.9763
**24**	−1.131	12.985	0.9193	**57**	−0.540	6.358	0.9690
**25**	−0.692	7.917	0.9672	**58**	−0.180	2.073	0.9521
**26**	−0.420	4.868	0.9733	**59**	−0.185	2.118	0.9609
**27**	−0.639	7.247	0.9662	**60**	−0.157	1.820	0.9465
**28**	−0.461	5.307	0.9593	**61**	−0.241	2.856	0.9792
**29**	−0.382	4.464	0.9659	**62**	−0.175	2.051	0.9765
**30**	−0.451	5.916	0.9656	**63**	−0.193	2.343	0.9682
**31**	−0.301	3.477	0.9657	**64**	−0.151	1.802	0.9488
**32**	−0.287	3.322	0.9707	**65**	−0.132	1.550	0.9358
**33**	−0.479	5.531	0.9187				

**Table 3:** Characteristics of the tested compounds.

No	log (*k*_m_/*K*_AM_)	log *BB*	log BB* [75]	*TPSA* Å^2^	*HBD*	*HBA*	*NRB*	*MW* g mol^−1^	*α* Å^3^	*Ƥ* m^3^ mol^−1^
**1**	−0.957	0.117	0.21	48.27	0	5	2	242.28	27.55	497.81
**2**	−0.834	0.305	0.32	48.27	0	5	2	256.30	29.30	528.90
**3**	−0.950	0.226	0.26	48.27	0	5	2	276.72	29.37	526.66
**4**	−0.789	0.270	0.30	48.27	0	5	2	276.72	29.37	526.66
**5**	−0.780	0.209	0.24	48.27	0	5	2	276.72	29.37	526.66
**6**	−0.674	0.360	0.34	48.27	0	5	2	311.17	31.20	555.51
**7**	−1.099	−0.243	−0.16	74.57	0	7	4	286.29	30.27	561.26
**8**	−0.968	−0.051	−0.04	74.57	0	7	4	300.31	32.02	592.35
**9**	−1.140	−0.293	−0.28	83.80	0	8	5	316.31	32.57	611.52
**10**	−1.012	−0.167	−0.20	83.80	0	8	6	330.34	34.40	650.13
**11**	−0.920	−0.151	−0.12	74.57	0	7	4	320.73	32.09	590.11
**12**	−0.949	−0.132	−0.10	74.57	0	7	5	300.31	32.09	604.35
**13**	−0.819	0.055	0.02	74.57	0	7	5	314.38	33.85	635.45
**14**	−0.876	−0.167	−0.20	83.80	0	8	6	330.34	34.40	599.87
**15**	−0.771	0.029	0.00	74.57	0	7	5	334.76	33.92	630.96
**16**	−0.766	−0.033	−0.05	74.57	0	7	5	334.76	33.92	650.13
**17**	−0.660	0.117	0.05	74.57	0	7	5	369.20	35.74	628.72
**18**	−0.850	0.038	0.06	61.41	0	6	2	280.31	30.82	628.72
**19**	−0.845	0.225	0.17	61.41	0	6	2	294.34	33.57	657.57
**20**	−0.684	0.225	0.17	61.41	0	6	2	294.34	32.57	578.01
**21**	−0.833	0.417	0.29	61.41	0	6	2	308.37	34.33	609.11
**22**	−0.722	0.006	−0.04	70.64	0	7	3	310.34	33.12	597.18
**23**	−0.755	0.003	−0.05	70.64	0	7	3	310.34	33.12	597.18
**24**	−1.113	0.141	0.11	61.41	0	6	2	314.75	32.64	575.77
**25**	−0.899	0.194	0.16	61.41	0	6	2	314.75	32.64	575.77
**26**	−0.687	0.130	0.09	61.41	0	6	2	314.75	32.64	575.77
**27**	−0.860	0.281	0.19	61.41	0	6	2	349.20	34.47	604.62
**28**	−0.725	0.297	0.21	61.41	0	6	2	349.20	34.47	604.62
**29**	−0.650	0.227	0.15	48.27	0	5	2	290.32	33.92	604.97
**30**	−0.772	0.407	0.26	48.27	0	5	2	304.35	35.68	636.07
**31**	−0.541	0.407	0.26	48.27	0	5	2	304.35	35.68	636.07
**32**	−0.521	0.407	0.26	48.27	0	5	2	304.35	35.68	636.07
**33**	−0.743	0.188	0.05	57.50	0	6	3	320.35	36.23	655.23
**34**	−0.583	0.172	0.04	57.50	0	6	3	320.35	36.23	655.23
**35**	−0.566	0.298	0.11	57.50	0	6	4	334.41	38.05	693.84
**36**	−0.699	0.594	0.38	48.27	0	5	2	318.37	37.43	667.16
**37**	−0.693	0.331	0.20	48.27	0	5	2	324.76	35.75	633.82
**38**	−0.682	0.376	0.25	48.27	0	5	2	324.76	35.75	633.82
**39**	−0.522	0.319	0.19	48.27	0	5	2	324.76	35.75	633.82
**40**	−0.393	0.465	0.29	48.27	0	5	2	359.21	37.57	662.67
**41**	−0.615	0.341	0.22	48.27	0	5	3	304.35	35.75	643.58
**42**	−0.521	0.459	0.28	48.27	0	5	3	338.82	37.57	672.43
**43**	−0.469	0.459	0.28	48.27	0	5	3	338.82	37.57	672.43
**44**	−0.451	0.459	0.28	48.27	0	5	3	338.82	37.57	672.43
**45**	−0.474	0.524	0.33	48.27	0	5	3	318.41	37.57	674.68
**46**	−0.373	0.712	0.45	48.27	0	5	3	332.44	39.26	705.77
**47**	−0.342	0.712	0.45	48.27	0	5	3	332.44	39.26	705.77
**48**	−0.356	0.635	0.38	48.27	0	5	3	352.85	39.26	703.53
**49**	−0.389	0.635	0.38	48.27	0	5	3	352.85	39.33	703.53
**50**	−0.302	0.635	0.38	48.27	0	5	3	352.85	39.33	703.53
**51**	−0.531	0.424	0.19	57.50	0	6	5	348.44	39.88	732.46
**52**	−0.387	0.602	0.37	57.50	0	6	5	362.47	41.63	763.55
**53**	−0.416	0.526	0.23	57.50	0	6	5	382.88	41.70	761.31
**54**	−0.505	0.526	0.23	57.50	0	6	5	382.88	41.70	761.31
**55**	−0.343	0.526	0.22	57.50	0	6	5	382.88	41.70	761.31
**56**	−0.550	0.635	0.38	48.27	0	5	3	352.85	39.33	703.53
**57**	−0.803	0.440	0.27	48.27	0	5	3	338.82	37.57	672.43
**58**	−0.317	0.551	0.31	48.27	0	5	3	373.27	39.40	701.28
**59**	−0.326	0.551	0.31	48.27	0	5	3	373.27	39.40	701.28
**60**	−0.260	0.551	0.31	48.27	0	5	3	373.27	39.40	701.28
**61**	−0.456	0.459	0.29	48.27	0	5	4	318.37	37.58	682.19
**62**	−0.312	0.651	0.41	48.27	0	5	4	332.40	39.33	713.29
**63**	−0.370	0.567	0.34	48.27	0	5	4	352.82	39.40	711.04
**64**	−0.256	0.551	0.33	48.27	0	5	4	352.82	39.40	711.04
**65**	−0.190	0.701	0.43	48.27	0	5	4	387.26	41.22	739.89

**Table 4:** Statistics of the established models M1-M12, M5*, M11*, and M12*: the coefficient of determination (*R*^2^, *Q*^2^), the determination coefficient adjusted (*R*^2^_adj_), the determination coefficient predicted (*R*^2^_pred_), the predicted residual error sum of squares (*PRESS*), the variance inflation factor (*VIF*)*,* the sum of squared differences from the mean (*SS*), the mean squared error (*MSE*), *F*-value, *p*-value; * the highest value.

Model	*R* ^2^	*R* ^2^_adj_	*R* ^2^_pred_	*PRESS*	VIF*	*SS*	*MSE*	*F*	*p*	*Q*^2^LOO	PRESS_LOO_
M1: log *BB* vs. (log (*k*_m_/*K*_AM_), *TPSA*, *NRB*, *α*)	0.9202	0.9149	0.9088	0.3889	5.3	4.2636	0.0057	173.0	0.00000	–	–
M2: log *BB* vs. (log (*k*_m_/*K*_AM_), *TPSA*, *NRB*, *Ƥ*)	0.9070	0.9008	0.8931	0.4556	4.7	4.2636	0.0066	146.3	0.00000	–	–
M3: log *BB* vs. (log (*k*_m_/*K*_AM_), *TPSA*, *NRB*, *MW*)	0.8943	0.8873	0.8799	0.5119	5.0	4.2636	0.0075	129.9	0.00000	–	–
M4: log *BB* vs. (log (*k*_m_/*K*_AM_), *HBA*, *NRB*, *α*)	0.9260	0.9210	0.9150	0.3625	5.4	4.2636	0.2774	187.6	0.00000	–	–
M5: log *BB* vs. (log (*k*_m_/*K*_AM_), HBA, *NRB*, *Ƥ*)	0.9109	0.9049	0.8971	0.4385	4.7	4.2636	0.0063	153.3	0.00000	0.9109	0.4385
M6: log *BB* vs. (log (*k*_m_/*K*_AM_), *HBA*, *NRB*, *MW*)	0.8914	0.8841	0.8764	0.5268	4.8	4.2636	0.0077	123.1	0.00000	–	–
M7: log BB* vs. (log (*k*_m_/*K*_AM_), *TPSA*, *NRB*, *α*)	0.8523	0.8425	0.8310	0.3247	5.3	1.9212	0.0047	86.6	0.00000	–	–
M8: log BB* vs. (log (*k*_m_/*K*_AM_), *TPSA*, *NRB*, *Ƥ*)	0.8508	0.8409	0.8297	0.3271	4.7	1.9212	0.0048	85.5	0.00000	–	–
M9: log BB* vs. (log (*k*_m_/*K*_AM_), *TPSA*, *NRB*, *MW*)	0.8529	0.8430	0.8324	0.3219	5.0	1.9212	0.0047	86.9	0.00000	–	–
M10: log BB* vs. (log (*k*_m_/*K*_AM_), *HBA*, *NRB*, *α*)	0.8682	0.8595	0.8489	0.2904	5.4	1.9212	0.0042	98.8	0.00000	–	–
M11: log BB* vs. (log (*k*_m_/*K*_AM_), HBA, *NRB*, *Ƥ*)	0.8659	0.8569	0.8466	0.2941	4.7	1.9212	0.0043	96.8	0.00000	0.8659	0.2947
M12: log BB* vs. (log (*k*_m_/*K*_AM_), *HBA*, *NRB*, *MW*)	0.8662	0.8572	0.8473	0.2933	4.8	1.9211	0.0043	97.1	0.00000	0.8662	0.2933
M5*: log *BB* vs. (log (*k*_m_/*K*_AM_), HBA, *Ƥ*)	0.9087	0.9042	0.8994	0.4290	4.7	4.2636	0.2178	202.4	0.00000	0.9087	0.4290
M11*: log BB* vs. (log (*k*_m_/*K*_AM_), HBA, *Ƥ*)	0.8656	0.8590	0.8513	0.2858	4.7	1.9212	0.0042	131.0	0.00000	0.8656	0.2858
M12*: log BB* vs. (log (*k*_m_/*K*_AM_), *HBA*, *MW*)	0.8661	0.8595	0.8516	0.2852	4.1	1.9212	0.0042	131.5	0.00000	0.8515	0.2852

## Data Availability

The data presented in this study are available on request from the corresponding author. The samples of the investigated compounds are available from the corresponding author.

## References

[1] Eriksson L., Jaworska J., Worth A.P., Cronin M.T., McDowell R.M., Gramatica P. (2003). Methods for reliability and uncertainty assessment and for applicability evaluations of classification and regression based QSARs. Environ. Health Perspect..

[2] Liu P., Long W. (2009). Current mathematical methods used in QSAR/QSPR studies. Int. J. Mol. Sci..

[3] Iyer M., Mishra R., Han Y., Hopfinger A.J. (2002). Predicting blood-brain barrier partitioning of organic molecules using membrane-interaction QSAR analysis. Pharm. Res..

[4] Adenot M., Lahana R. (2004). Blood-brain barrier permeation models: Discriminating between potential CNS and non-CNS drugs including P-glycoprotein substrates. J. Chem. Inf. Comput. Sci..

[5] de Campos L.J., de Melo E.B. (2014). Modeling structure–activity relationships of prodiginines with antimalarial activity using GA/MLR and OPS/PLS. J. Mol. Graph. Model..

[6] Kimani N.M., Matasyoh J.C., Kaiser M., Nogueira M.S., Trossini G.H.G., Schmidt T.J. (2018). Complementary quantitative structure–activity relationship models for the antitrypanosomal activity of sesquiterpene lactones. Int. J. Mol. Sci..

[7] Pourbasheer E., Riahi S., Ganjali M.R., Norouzi P. (2010). Quantitative structure–activity relationship (QSAR) study of interleukin-1 receptor associated kinase 4 (IRAK-4) inhibitor activity by the genetic algorithm and multiple linear regression (GA-MLR) method. J. Enzyme Inhib. Med. Chem..

[8] Tugcu G., Saçan M.T., Vračko M., Novič M., Minovski N. (2012). QSTR modelling of the acute toxicity of pharmaceuticals to fish. SAR QSAR Environ. Res..

[9] Ciura K., Belka M., Kawczak P., Bączek T., Markuszewski M.J., Nowakowska J. (2017). Combined computational-experimental approach to predict blood–brain barrier (BBB) permeation based on “green” salting-out thin layer chromatography supported by simple molecular descriptors. J. Pharm. Biomed. Anal..

[10] Liu R., Sun H., So S.-S. (2001). Development of quantitative structure-property relationship models for early ADME evaluation in drug discovery. 2. Blood-brain barrier penetration. J. Chem. Inf. Comput. Sci..

[11] Ajay, Bemis G.W., Murcko M.A. (1999). Designing libraries with CNS activity. J. Med. Chem..

[12] Kouskoura M.G., Piteni A.I., Markopoulou C.K. (2019). A new descriptor via bio-mimetic chromatography and modeling for the blood brain barrier (Part II). J. Pharm. Biomed. Anal..

[13] Goldstein G.W., Betz A.L. (1986). The blood-brain barrier. Sci. Am..

[14] Pardridge W.M. (1998). CNS drug design based on principles of blood brain barrier transport. J. Neurochem..

[15] Begley D.J. (1996). The blood-brain barrier: Principles for targeting peptides and drugs to the central nervous system. J. Pharm. Pharmacol..

[16] Mouritsen O.G., Jorgensen K. (1998). A new look at lipid membrane structure in relation to drug research. Pharm. Res..

[17] Sugano K., Kansy M., Artursson P., Avdeef A., Bendels S., Di L., Ecker G.F., Faller B., Fischer H., Gerebtzoff G. (2010). Coexistence of passive and carrier-mediated processes in drug transport. Nat. Rev. Drug Discov..

[18] Sugiyama Y., Kusuhara H., Suzuki H. (1999). Kinetic and biochemical analysis of carrier-mediated efflux of drugs through the blood-brain and blood-cerebrospinal fluid barriers: Importance in the drug delivery to the brain. J. Cont. Rel..

[19] Seddon A.M., Casey D., Law R.V., Gee A., Templera R.H., Cesab O. (2009). Drug interactions with lipid membranes. Chem. Soc. Rev..

[20] Wolak D.J., Thorne R.G. (2013). Diffusion of macromolecules in the brain: Implications for drug delivery. Mol. Pharm..

[21] Banks W.A. (2009). Characteristics of compounds that cross the blood-brain barrier. BMC Neurol..

[22] Hemmateenejad B., Miri R., Safarpour M.A., Mehdipour A.R. (2006). Accurate prediction of the blood-brain partitioning of a large set of solutes using ab initio calculations and genetic neural network modeling. J. Comput. Chem..

[23] Winkler D.A., Burden F.R. (2004). Modeling blood–brain barrier partitioning using Bayesian neural nets. J. Mol. Graphics Model..

[24] Luco M. (1999). Prediction of the brain–blood distribution of a large set of drugs from structurally derived descriptors using partial least-squares (PLS) modeling. J. Chem. Inf. Comput. Sci..

[25] Liu X., Tu M., Kelly R.S., Chen C., Smith B.J. (2004). Development of a computational approach to predict blood-brain barrier permeability. Drug Metab. Dispos..

[26] Lombardo F., Blake J.F., Curatolo W. (1996). Computation of brain-blood partitioning of organic solutes via free-energy calculations. J. Med. Chem..

[27] Keserü G.M., Molnar L. (2001). High-throughput prediction of blood-brain partitioning: A thermodynamic approach. J. Chem. Inf. Comput. Sci..

[28] Norinder U., Sjőberg P., Osterberg T. (1998). Theoretical calculations and prediction of brain-blood partitioning of organic solutes using MolSurf parametrization and PLS statistics. J. Pharm. Sci..

[29] Crivori P., Cruciani G., Carrupt P.-A., Testa B. (2000). Predicting blood-brain permeation from three-dimensional molecular structure. J. Med. Chem..

[30] Osterberg T., Norinder U. (2000). Prediction of polar surface area and drug transport processes using simple parameters and PLS statistics. J. Chem. Inf. Comput. Sci..

[31] Rose K., Hall L.H., Kier L.B. (2002). Modeling blood-brain barrier partitioning using the electrotopological state. J. Chem. Inf. Comput. Sci..

[32] Basak S.C., Gute B.D., Drewes L.R. (1996). Predicting blood brain transport of drugs: A computational approach. Pharm. Res..

[33] Kelder J., Grootenhuis P.D., Bayada D.M., Delbressine L.P.C., Ploemen J.P. (1999). Polar molecular surface as dominating determinant for oral absorption and brain penetration of drugs. Pharm. Res..

[34] Ertl P., Rohde B., Selzer P. (2000). Fast calculation of molecular polar surface area as a sum of fragment based contributions and its application to the prediction of drug transport properties. J. Med. Chem..

[35] Abraham M.H., Chadha H.S., Mitchell R.C. (1994). Hydrogen bonding. 33. Factors that influence the distribution of solutes between blood and brain. J. Pharm. Sci..

[36] van de Waterbeemd H., Camenish G., Folkers G., Chretien J.R., Raevsky O.A. (1998). Estimation of blood-brain barrier crossing of drugs using molecular size and shape, and H-bonding descriptors. J. Drugs Target..

[37] Kaliszan R. (2007). QSRR Quantitative structure—(chromatographic) retention relationships. Chem. Rev..

[38] Dąbrowska M., Komsta Ł., Krzek J., Kokoszka K. (2015). Lipophilicity study of eight cephalosporins by reversed-phase thin-layer chromatographic method. Biomed. Chromatogr..

[39] Kempińska D., Chmiel T., Kot-Wasik A., Mróz A., Mazerska Z., Namieśnik J. (2019). State of the art and prospects of methods for determination of lipophilicity of chemical compounds. TrAC Trends Anal. Chem..

[40] Kurbatova S.V., Saifutdinov B.R., Larionov O.G., Meshkovaya V.V. (2009). The influence of the structure of some aromatic heterocyclic derivatives on their retention in reversed-phase high-performance liquid chromatography. Russ. J. Phys. Chem..

[41] Sagandykova G.N., Pomastowski P.P., Kaliszan R., Buszewski B. (2018). Modern analytical methods for consideration of natural biological activity. TrAC Trends Anal. Chem..

[42] Milošević N.P., Stojanović S.Z., Penov-Gaši K., Perišić-Janjić N., Kaliszan R. (2014). Reversed- and normal-phase liquid chromatography in quantitative structure retention-property relationships of newly synthesized seco-androstene derivatives. J. Pharm. Biomed. Anal..

[43] Héberger K. (2007). Quantitative structure-(chromatographic) retention relationships. J. Chromatogr. A.

[44] Andrić F., Héberger K. (2015). Towards better understanding of lipophilicity: Assessment of in silico and chromatographic log P measures for pharmaceutically important compounds by nonparametric rankings. J. Pharm. Biomed. Anal..

[45] Valko K., Nunhuck S., Bevan C., Abraham M.H., Reynolds D.P. (2003). Fast gradient HPLC method to determine compounds binding to human serum albumin. Relationships with octanol/water and immobilized artificial membrane lipophilicity. J. Pharm. Sci..

[46] Valko K. (2016). Lipophilicity and biomimetic properties measured by HPLC to support drug discovery. J. Pharm. Biomed. Anal..

[47] Ciura K., Dziomba S. (2020). Application of separation methods for in vitro prediction of blood–brain barrier permeability—The state of the art. J. Pharm. Biomed. Anal..

[48] Milošević N., Janjić N., Milić N., Milanović M., Popović J., Antonowić D. (2014). Pharmacokinetics and toxicity predictors of new s-triazines, herbicide candidates, in correlation with chromatographic retention constants. J. Agric. Food Chem..

[49] Tsopelas F., Giaginis C., Tsantili-Kakoulidou A. (2017). Lipophilicity and biomimetic properties to support drug discovery. Expert Opin. Drug Discov..

[50] Russo G., Grumetto L., Szucs R., Barbato F., Lynen F. (2018). Screening therapeutics according to their uptake across the blood-brain barrier: A high throughput method based on immobilized artificial membrane liquid chromatography-diode-array-detection coupled to electrospray-time-of-flight mass spectrometry. Eur. J. Pharm. Biopharm..

[51] Stergiopoulos C., Tsopelas F., Valko K., Ochsenkühn-Petropoulou M. (2022). The use of biomimetic chromatography to predict acute aquatic toxicity of pharmaceutical compounds. Toxicol. Environ. Chem..

[52] Escuder-Gilabert L., Molero-Monfort M., Villanueva- Camañas R.M., Sagrado S., Medina-Hernández M.J. (2004). Potential of biopartitioning micellar chromatography as an in vitro technique for predicting drug penetration across the blood-brain barrier. J. Chromatogr. B Analyt. Technol. Biomed. Life Sci..

[53] Ruiz-Ángel M., Garcia-Álvarez-Coque M., Berthod A. (2009). New insights and recent developments in micellar liquid chromatography. Sep. Pur. Rev..

[54] Rambla-Alegre M. (2012). Basic principles of MLC. Chromatogr. Res. Int..

[55] Kalyankar T.M., Kulkarni P.D., Wadher S.J., Pekamwar S.S. (2014). Applications of micellar liquid chromatography in bioanalysis: A review. J. Appl. Pharm. Sci..

[56] Tsopelas F., Danias P., Pappa A., Tsantili-Kakoulidou A. (2020). Biopartitioning micellar chromatography under different conditions: Insight into the retention mechanism and the potential to model biological processes. J. Chromatogr. A.

[57] Foley J.P. (1990). Critical compilation of solute-micelle binding constants and related parameters from micellar liquid chromatographic measurements. Anal. Chim. Acta.

[58] Janicka M., Sztanke M., Sztanke K. (2013). Reversed-phase liquid chromatography with octadecylsilyl, immobilized artificial membrane and cholesterol columns in correlation studies with in silico biological descriptors of newly synthesized antiproliferative and analgesic active compounds. J. Chromatogr. A.

[59] Sztanke M., Tuzimski T., Janicka M., Sztanke K. (2015). Structure-retention behaviour of biologically active fused 1,2,4-triazinones—Comparison with in silico molecular properties. Eur. J. Pharm. Sci..

[60] Sztanke M., Rzymowska J., Janicka M., Sztanke K. (2019). Synthesis, structure confirmation, identification of in vitro antiproliferative activities and correlation of determined lipophilicity parameters with in silico bioactivity descriptors of two novel classes of fused azaisocytosine-like congeners. Arabian J. Chem..

[61] Sztanke M., Sztanke K. (2017). 3-(2-Phenylethyl)-8-aryl-7,8-dihydroimidazo[2,1-c][1,2,4]triazin-4(6H)-ones, Method for Obtaining Them and Medical Application. Polish Patent.

[62] Sztanke M., Sztanke K. (2017). 3-Ethyl-8-aryl-7,8-dihydroimidazo[2,1-c][1,2,4]triazin-4(6H)-ones, Method for Obtaining Them and Medical Application. Polish Patent.

[63] Sztanke K., Sztanke M. (2015). Ethyl 2-(4-oxo-4,6,7,8-tetrahydroimidazo[2,1-c][1,2,4]triazin-3-yl)acetates, Method for Obtaining Them and Medical Application. Polish Patent.

[64] Sztanke M., Rzymowska J., Sztanke K. (2013). Synthesis, structure elucidation and in vitro anticancer activities of novel derivatives of diethyl (2E)-2-[(2E)-(1-arylimidazolidin-2-ylidene)hydrazono]succinate and ethyl(4-oxo-8-aryl-4,6,7,8-tetrahydroimidazo[2,1-*c*][1,2,4]triazin-3-yl)acetate. Bioorg. Med. Chem..

[65] Sztanke M., Sztanke K., Rajtar B., Świątek Ł., Boguszewska A., Polz-Dacewicz M. (2019). The influence of some promising fused azaisocytosine-containing congeners on zebrafish (*Danio rerio*) embryos/larvae and their antihaemolytic, antitumour and antiviral activities. Eur. J. Pharm. Sci..

[66] Sztanke K., Tuzimski T., Sztanke M., Rzymowska J., Pasternak K. (2011). Synthesis, structure elucidation, determination of the lipophilicity and identification of antitumour activities in vitro of novel 3-(2-furanyl)-8-aryl-7,8-dihydroimidazo[2,1-*c*][1,2,4]triazin-4(6*H*)-ones with a low cytotoxicity towards normal human skin fibroblast cells. Bioorg. Med. Chem..

[67] Sztanke K., Sztanke M., Pasternak K. (2012). 3-(2-Furanyl)-7,8-dihydroimidazo[2,1-c][1,2,4]triazin-4(6H)-ones Substituted with Mono- or Dichlorophenyl and Process for the Preparation Thereof. Polish Patent.

[68] Sztanke K., Sztanke M., Pasternak K. (2012). Derivatives of 3-(2-furanyl)-7,8-dihydroimidazo[2,1-c][1,2,4]triazin-4(6H)-one Substituted with Phenyl, Alkylphenyl, Alkoxyphenyl and Process for the Preparation Thereof. Polish Patent.

[69] Sztanke K. (2008). New 8-aryl-3-phenyl-6,7-dihydro-4H-imidazo[2,1-c][1,2,4]triazine-4-ones and Methods for Their Manufacture. Polish Patent.

[70] Sztanke K., Pasternak K., Sztanke M., Kandefer-Szerszeń M., Kozioł A.E., Dybała I. (2009). Crystal structure, antitumour and antimetastatic activities of disubstituted fused 1,2,4-triazinones. Bioorg. Med. Chem. Lett..

[71] Tuzimski T., Sztanke K. (2005). Retention data for some carbonyl derivatives of imidazo[2,1-*c*][1,2,4]triazine in reversed-phase systems in TLC and HPLC and their use for determination of lipophilicity. Part 1. Lipophilicity of 8-aryl-3-phenyl-6,7-dihydro-4*H*-imidazo[2,1-*c*][1,2,4]triazin-4-ones. J. Planar Chromatogr..

[72] Bartyzel A., Sztanke M., Sztanke K. (2017). Thermal behaviour of antiproliferative active 3-(2-furanyl)-8-aryl-7,8-dihydroimidazo[2,1-*c*][1,2,4]triazin-4(6*H*)-ones. J. Therm. Anal. Calorim..

[73] Stępniowska A., Sztanke M., Tuzimski T., Korolczuk M., Sztanke K. (2017). A simple stripping voltammetric method for the determination of a new anticancer prodrug in serum. Biosens. Bioelectron..

[74] Kozak J., Tyszczuk-Rotko K., Sadok I., Sztanke K., Sztanke M. (2022). Application of screen-printed sensor modified with carbon nanofibers for the voltammetric analysis of an anticancer disubstituted fused triazinone. Int. J. Mol. Sci..

[75] Janicka M., Sztanke M., Sztanke K. (2020). Predicting the blood-brain barrier permeability of new drug-like compounds via HPLC with various stationary phases. Molecules.

[76] Janicka M., Śliwińska A. (2022). Quantitative retention (structure)—Activity relationships in predicting the pharmaceutical and toxic properties of potential pesticides. Molecules.

[77] Lipinski C.A., Lombardo F., Dominy B.W., Feeney P.J. (1997). Experimental and computational approaches to estimate solubility and permeability in drug discovery and development settings. Adv. Drug Deliv. Rev..

[78] Clark D.E. (2003). In silico prediction of blood−brain barrier permeation. Drug Discov. Today.

[79] Roy K., Kar S., Das R.N. (2015). A Primer on QSAR/QSPR Modeling: Fundamental Concepts.

[80] Roy K., Kar S., Das R.N. (2015). Understanding the Basics of QSAR for Applications in Pharmaceutical Sciences and Risk Assessment.

[81] Hamadache M., Benkortbi O., Hanini S., Amrane A. (2018). QSAR modeling in ecotoxicological risk assessment: Application to the prediction of acute contact toxicity of pesticides on bees (*Apis mellifera* L.). Environ. Sci. Pollut. Res..

[82] Hamadache M., Benkortbi O., Hanini H., Amrane A., Khaouane L., Si Moussa C. (2016). A quantitative structure activity relationship for acute oral toxicity of pesticides on rats: Validation, domain of application and prediction. J. Hazard. Mater..

[83] Sawant S.D., Nerkar A.G., Pawar N.D., Velapure A.V. (2014). Design, synthesis, QSAR studies and biological evaluation of novel triazolopiperazine based β-amino amides as dipeptidyl peptidase-IV (DPP-IV) inhibitors: Part-II. Int. J. Pharm. Pharm. Sci..

[84] Clementi M., Clementi S., Fornaciari M., Orlandi F., Romano B. (2001). The GOLPE procedure for predicting olive crop production from climatic parameters. J. Chemom..

[85] Chen J.W., Li X.H., Yu H.Y., Wang Y.N., Qiao X.L. (2008). Progress and perspectives of quantitative structure–activity relationships used for ecological risk assessment of toxic organic compounds. Sci. China Ser. B-Chem..

[86] (2007). Organization for Economic Co-Operation and Development, Guidance Document on the Validation of (Quantitative) Structure–Activity Relationship[(Q)SAR] Models. https://www.oecd.org/env/guidance-document-on-the-validation-of-quantitative-structure-activity-relationship-q-sar-models-9789264085442-en.htm.

[87] Saaidpour S., Bahmani A., Rostami A. (2015). Prediction the normal boiling points of primary, secondary and tertiary liquid amines from their molecular structure descriptors. CMST.

[88] Kaliszan R., Markuszewski M. (1996). Brain-blood distribution described by a combination of partition coefficients and molecular mass. Int. J. Pharm..

[89] Testa B., Crivori P., Reist M., Carrupt P.A. (2000). The influence of lipophilicity on the pharmacokinetic behavior of drugs: Concepts and examples. Persp. Drug Discov. Des..

[90] Levin V.A. (1980). Relationship of octanol/water partition coefficient and molecular weight to rat brain capillary permeability. J. Med. Chem..

[91] Platts J.A., Abraham M.H., Hersey A., Butina D. (2000). Estimation of molecular linear free energy relationship descriptors. 4. Correlation and prediction of cell permeation. Pharm. Res..

[92] Janicka M., Mycka A., Sztanke M., Sztanke K. (2021). Predicting pharmacokinetic properties of potential anticancer agents via their chromatographic behavior on different reversed phase materials. Int. J. Mol. Sci..

[93] Pan D., Iyer M., Liu J., Li Y., Hopfinger A.J. (2004). Constructing optimum blood brain barrier QSAR models using a combination of 4D-molecular similarity measures and cluster analysis. J. Chem. Inf. Comput. Sci..

[94] Veber D.F., Johnson S.R., Cheng H.-Y., Smith B.R., Ward K.W., Kopple K.D. (2002). Molecular properties that influence the oral bioavailability of drug candidates. J. Med. Chem..

